# Selective Antagonism of Lactiplantibacillus plantarum and Pediococcus acidilactici against *Vibrio* and *Aeromonas* in the Bacterial Community of Artemia nauplii

**DOI:** 10.1128/spectrum.00533-23

**Published:** 2023-07-10

**Authors:** Weihua Xu, Zhaolin Lv, Qingqi Guo, Zhaojie Deng, Canmin Yang, Zhaozhao Cao, Yi Li, Cuifen Huang, Zizhan Wu, Shijun Chen, Yuhui He, Jijia Sun, Yiying Liu, Lian Gan

**Affiliations:** a College of Marine Sciences, South China Agricultural University, Guangzhou, China; b Nansha-South China Agricultural University Fishery Research Institute, Guangzhou, China; c Guangdong Laboratory for Linnan Modern Agriculture, Guangzhou, China; d University Joint Laboratory of Guangdong Province, Hong Kong and Macao Region on Marine Bioresource Conservation and Exploitation, Guangzhou, China; Institut Pasteur

**Keywords:** selective antagonism, probiotics, microbiota community, pathogens, *Artemia*

## Abstract

Empiric probiotics are commonly consumed by healthy individuals as a means of disease prevention, pathogen control, etc. However, controversy has existed for a long time regarding the safety and benefits of probiotics. Here, two candidate probiotics, Lactiplantibacillus plantarum and Pediococcus acidilactici, which are antagonistic to *Vibrio* and *Aeromonas* species *in vitro*, were tested on *Artemia* under *in vivo* conditions. In the bacterial community of Artemia nauplii, *L. plantarum* reduced the abundance of the genera *Vibrio* and *Aeromonas* and *P. acidilactici* significantly increased the abundance of *Vibrio* species in a positive dosage-dependent manner, while higher and lower dosages of *P. acidilactici* increased and decreased the abundance of the genus *Aeromonas*, respectively. Based on the liquid chromatography-mass spectrometry (LC-MS) and gas chromatography-mass spectrometry (GC-MS) analyses of the metabolite of *L. plantarum* and *P. acidilactici*, pyruvic acid was used in an *in vitro* test to explain such selective antagonism; the results showed that pyruvic acid was conducive or suppressive to V. parahaemolyticus and beneficial to A. hydrophila. Collectively, the results of this study demonstrate the selective antagonism of probiotics on the bacterial community composition of aquatic organisms and the associated pathogens.

**IMPORTANCE** Over the last decade, the common preventive method for controlling potential pathogens in aquaculture has been the use of probiotics. However, the mechanisms of probiotics are complicated and mostly undefined. At present, less attention has been paid to the potential risks of probiotic use in aquaculture. Here, we investigated the effects of two candidate probiotics, *L. plantarum* and *P. acidilactici*, on the bacterial community of Artemia nauplii and the *in vitro* interactions between these two candidate probiotics and two pathogens, *Vibrio* and *Aeromonas* species. The results demonstrated the selective antagonism of probiotics on the bacterial community composition of an aquatic organism and its associated pathogens. This research contributes to providing a basis and reference for the long-term rational use of probiotics and to reducing the inappropriate use of probiotics in aquaculture.

## INTRODUCTION

Probiotics are defined as “Live microorganisms which when administered in adequate amounts confer a health benefit on the host” (1). In the past 20 years, probiotics have been extensively applied in the fields of human medicine, food, agricultural production, and aquaculture (2–5), which is attributed to their roles in regulating the balance of bacterial community, improving the water environment, etc. (6–7). Contradictorily, probiotics are not universally good as they may be useless and even harmful under certain circumstances (8–10), but these adverse effects associated with probiotic consumption may be underreported (11). Medical authorities, such as the European Food Safety Authority (12) or the U.S. Food and Drug Administration (13), have rejected approving probiotic formulations as medical intervention modalities. In addition, several studies have reported that probiotics might activate virulence gene expression in pathogens and endanger the health of the hosts (14). To avoid the risks of probiotics, some researchers have studied the beneficial effects of inactivated probiotics (15, 16).

*Aeromonas* and *Vibrio* are common pathogens in aquatic animals and have drawn much attention owing to their wide distribution and high virulence (17, 18). Moreover, consumption of raw or improperly cooked seafood contaminated with pathogenic bacteria, such as Aeromonas hydrophila, Vibrio vulnificus, and Vibrio parahaemolyticus, can cause foodborne diseases: e.g., gastroenteritis, traumatic wound infections and septicemia (19, 20). To date, one of the most universal preventive methods to control potential pathogens in aquaculture is the use of probiotics (21). In aquaculture, probiotics are defined as “a live, dead or component of a microbial cell, which is administered via the feed or to the rearing water, benefiting the host by improving disease resistance, health status, growth performance, feed utilization, stress response or general vigor” (22). Lactic acid bacteria (LAB), including Lactiplantibacillus plantarum and Pediococcus acidilactici, are the major species of probiotics because they are “generally recognized as safe” (GRAS) microorganisms and have been well known for their ability in terms of the production of antibacterial substances (23, 24). Some studies have shown that *L. plantarum* promotes digestion, enhances growth performance, enhances immune response, and maintains the balance of intestinal flora in fish and crustaceans (25, 26). Moreover, it has been reported that *P. acidilactici* cells as additives in animal feed improve the bactericidal activity of zebrafish against the pathogens A. hydrophila and Vibrio anguillarum (27), and Bactocell (*P. acidilactici* CNCM I-4622) is the first commercially authorized bacterium approved for shrimp aquaculture. However, the latest research shows that “probiotics might be selectively beneficial or detrimental to pathogens” (26). To reduce the safety risk of probiotics and achieve by means of their improvement of human and aquatic animal well-being, a comprehensive assessment of probiotic effects on the host and in-depth research of the probiotic-pathogen interaction are necessary.

Artemia nauplii is a live food widely used in the larval culture of fish and crustaceans (28). Some researchers have indicated that bacterial pathogens infect fish and crustaceans mainly through the food chain and especially during the feeding with Artemia nauplii. Probiotics are capable of preventing colonization of bacterial pathogens during *Artemia* culture (29). Therefore, Artemia nauplii is often used as a model organism for studying the probiotic-pathogen interaction. Here, we investigated the *in vitro* interactions between two candidate probiotics, *L. plantarum* and *P. acidilactici*, and two pathogens, *Vibrio* and *Aeromonas* species, and the effects of two candidate probiotics on the bacterial community of Artemia nauplii. The results will provide reference to standardize probiotic assessments, utilization, and production.

## RESULTS

### Molecular identification and biological characterizations of lactic acid bacterial isolates.

**FIG 1:**
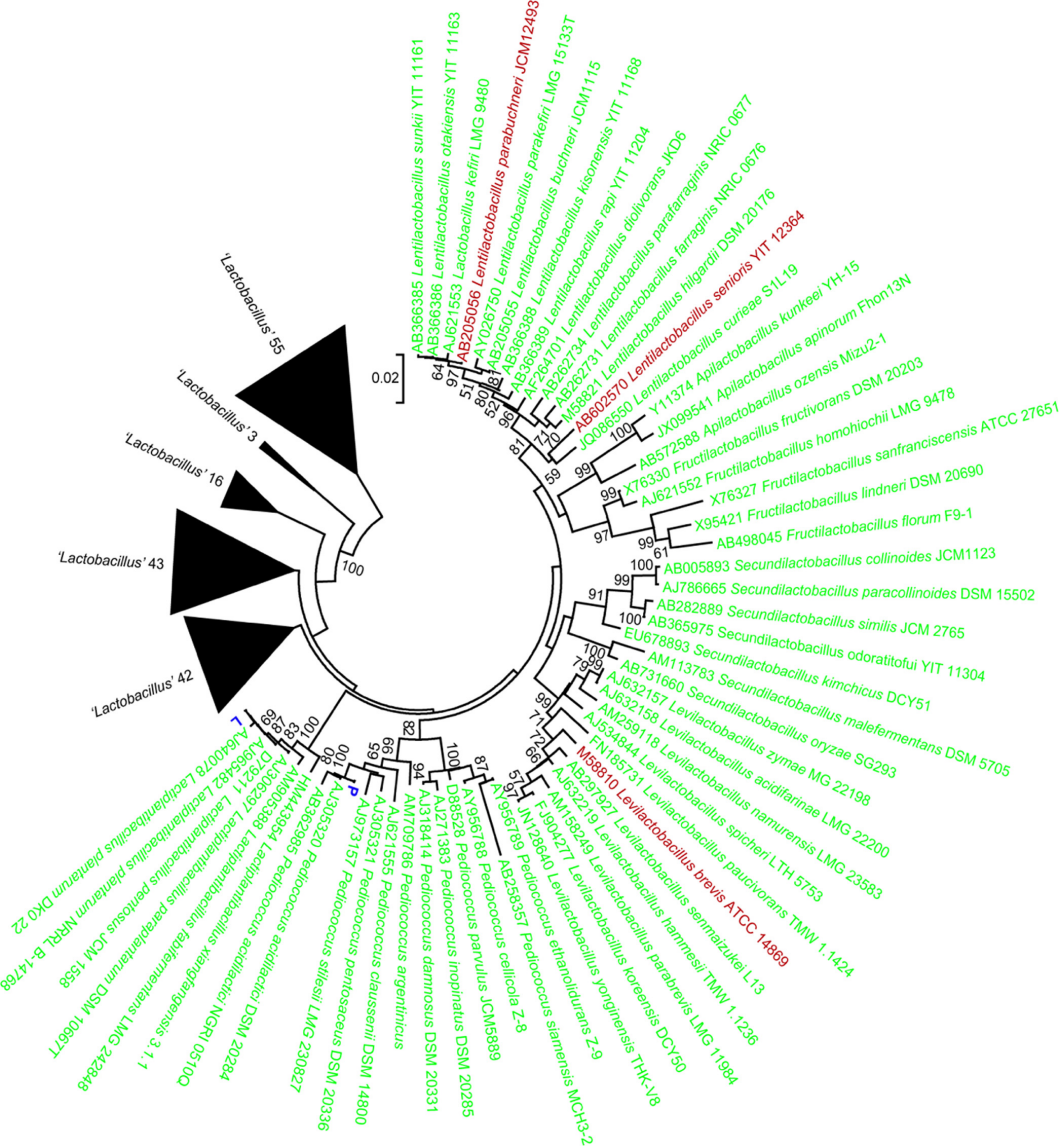
16S rRNA gene-based phylogenetic characterization of *L. plantarum* and *P. acidilactici*. The Phylogenetic tree displays 16S rRNA gene sequences (≥1,200 bp) of two LAB isolates in crustaceans from the South China Sea and 217 LAB type strains with good sequence quality downloaded from the National Centre for Biotechnology Information (NCBI) database website (http://www.ncbi.nlm.nih.gov/). The phylogenetic analyses were performed in Mega 7 using the Kimura two-parameter method with Gamma distribution (gamma parameter of 0.21) to calculate the evolutionary distances (86, 87). The bootstrap values represented at the nodes are based on 2,000 bootstrap replicates (88). The scale bar means an evolutionary distance of 0.02 nucleotide substitution per sequence position. Branch values lower than 50% are hidden. The accession number is followed by the name of each type strain. Blue, red, and green indicate the isolates of this study and the strains from human-related and terrestrial/plant sources of isolation. The term “*Lactobacillus*” represents the former genus name, which has been reclassified into 25 genera as proposed by Zheng and colleagues (91).

According to the results of 16S rRNA gene sequencing, the LAB isolates were identified as *L. plantarum* and *P. acidilactici* (Fig. 1). The motility and enzymatic characterization of *L. plantarum* and *P. acidilactici* are shown in Table 1: they exhibited amylase activity, and only *L. plantarum* displayed protease activity. For swarming, swimming, lipase, feed degradation, shrimp shell degradation, shrimp feces degradation, and shrimp muscle degradation assay, two LAB isolates showed no activity.

**TABLE 1:** Motility and enzymatic characterization of *L. plantarum* and *P. acidilactici*

Activity	Result for^*a*^:	
	*L. plantarum*	*P. acidilactici*
Swimming/swarming	−	−
Lipase	−	−
Amylase	+	+
Protease	+	−
Feed degradation	−	−
Shrimp shell degradation	−	−
Shrimp feces degradation	−	−
Shrimp muscle degradation	−	−

a + and − represent positive and negative for the corresponding activity, respectively.

### Antagonism of LAB isolates against the *Vibrio* and *Aeromonas* species.

**FIG 2:**
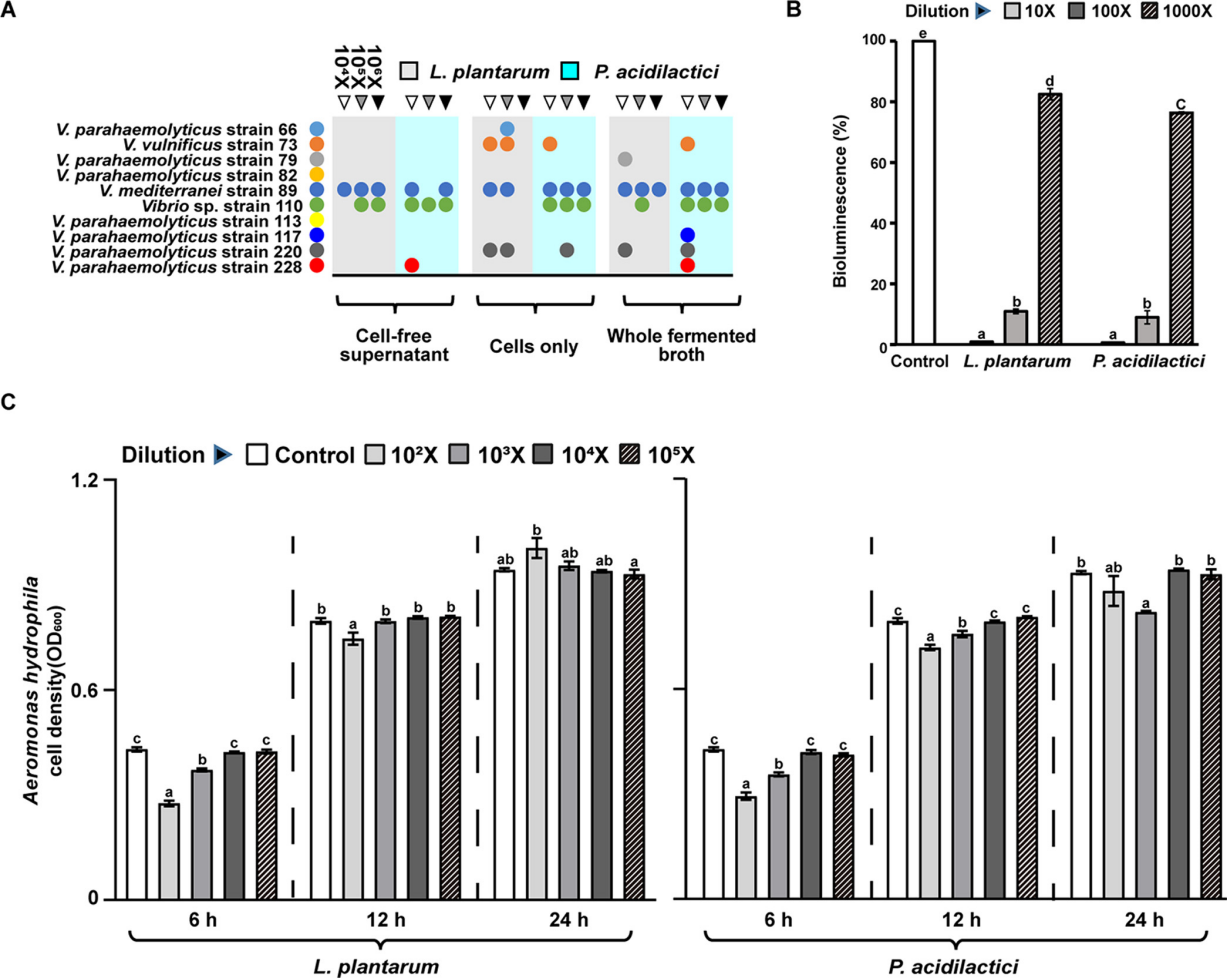
(A) Impacts of the cell-free supernatant, cells, and fermentation broths of *L. plantarum* and *P. acidilactici* on the proliferation of the 10 *Vibrio* strains. The fermentation broth, cells, and cell-free supernatant of each LAB isolate were diluted 10^4^-, 10^5^-, and 10^6^-fold; their impacts on the *Vibrio* species are indicated in the order of left, middle, and right of each column, respectively. The circles above the horizontal line meant the proliferation of the *Vibrio* species was inhibited by the corresponding cell-free supernatant, cells, and fermentation broths of two LAB isolates in the exponential growth phase within 24 h. (B) Impacts of the cell-free supernatant of *L. plantarum* and *P. acidilactici* on bioluminescence of type strain *V. campbellii* BB120. The cell-free supernatant of each LAB isolate was diluted 10-, 10^2^-, and 10³-fold. The bioluminescence of the control treatment was treated as 100%, and the other corresponding treatments were accordingly normalized. Statistical significance was evaluated by one-way ANOVA, followed by *post hoc* analysis with Duncan multiple-comparison test. The letters a to e indicate statistically significant differences (*P* < 0.05) based on a one-way ANOVA, followed by *post hoc* Duncan analysis. (C) Impacts of the fermentation broths of *L. plantarum* and *P. acidilactici* on the proliferation of A. hydrophila at 6, 12, and 24 h, respectively. The fermentation broth of each LAB isolate was diluted 10^3^-, 10^4^-, 10^5^-, and 10^6^-fold, and statistical significance was evaluated by one-way ANOVA, followed by *post hoc* analysis with Duncan multiple-comparison test. The letters a to d indicate statistically significant differences (*P* < 0.05). Each of the LAB treatments at different points in time was analyzed independently (indicated by dashed lines). Error bars represent the standard error of the mean (SEM) calculated from at least three biological replicates.

As shown in Fig. 2A, the cell-free supernatant, cells and fermentation broths of *L. plantarum* and *P. acidilactici* showed different levels of inhibition on various *Vibrio* species. The fermentation broth and cells of *L. plantarum* showed stronger inhibitory activity against *Vibrio* species than the cell-free supernatant of *L. plantarum.* Similarly, the fermentation broth of *P. acidilactici* presented stronger inhibitory activity against *Vibrio* species than the cell-free supernatant and cells of *P. acidilactici.* Moreover, the intermediate and high dosages of cell-free supernatant, cells, and fermentation broths of two LAB isolates displayed a more pronounced inhibitory effect against *Vibrio* species than those at low dosage. The impacts of the cell-free supernatants of *L. plantarum* and *P. acidilactici* on the bioluminescence of the type strain Vibrio campbellii BB120 are shown in Fig. 2B. The cell-free supernatant of two LAB isolates significantly suppressed the bioluminescence of *V. campbellii* BB120 in a positive dosage-dependent manner (*P* < 0.05). The impacts of the fermentation broths of *L. plantarum* and *P. acidilactici* on the proliferation of the Aeromonas hydrophila are displayed in Fig. 2C. The fermentation broth of the *L. plantarum* and *P. acidilactici* (diluted 10^2^-fold and 10^3^-fold) significantly inhibited the proliferation of the A. hydrophila (*P* < 0.05) at 6 h and at 6 and 12 h, respectively.

### The impacts of LAB isolates on TCBS-bacteria in *Artemia*.

**FIG 3:**
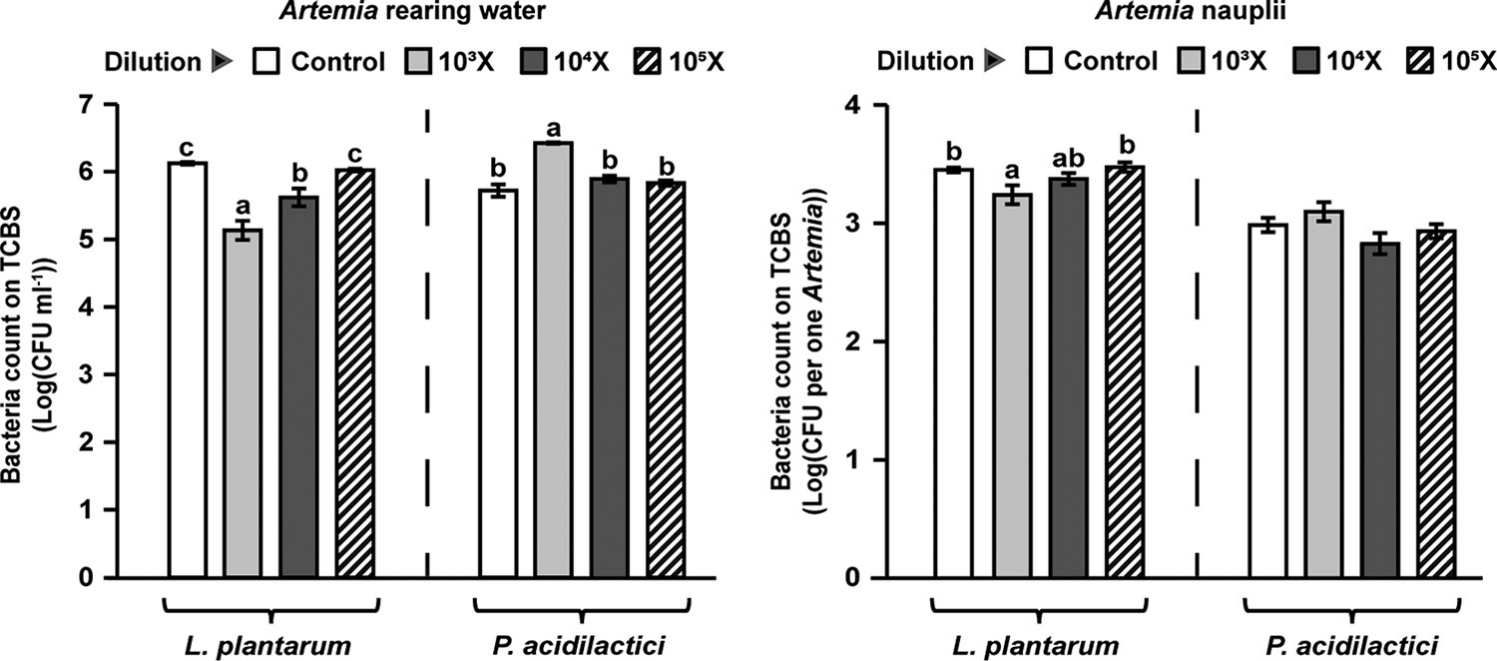
Impacts of the fermentation broths of *L. plantarum* and *P. acidilactici* on the TCBS-bacterial count in *Artemia* rearing water and Artemia nauplii. Statistical significance was evaluated by one-way ANOVA, followed by *post hoc* analysis with Duncan multiple-comparison test. The letters a to c indicate statistically significant differences (*P* < 0.05). Each LAB treatment was analyzed independently (indicated by dashed lines). Error bars represent the SEM calculated from at least nine biological replicates.

The impacts of the fermentation broths of *L. plantarum* and *P. acidilactici* on the count of TCBS (thiosulfate, citrate, bile salts, and sucrose)-cultured bacteria (TCBS-bacteria) in *Artemia* during hatching are shown in Fig. 3. In *Artemia* rearing water, *L. plantarum* significantly reduced the number of TCBS-bacteria in a positive dosage-dependent manner (*P* < 0.05). Contrarily, *P. acidilactici* (diluted 10^3^-fold) significantly increased the number of TCBS-bacteria (*P* < 0.05). In *Artemia* nauplii, *L. plantarum* (diluted 10^3^-fold) significantly reduced the number of TCBS-bacteria (*P* < 0.05), and there was no significant difference in the number of TCBS-bacteria by *P. acidilactici* treatments (*P* > 0.05).

### Impacts of LAB isolates on microbial community abundance in *Artemia*.

**FIG 4:**
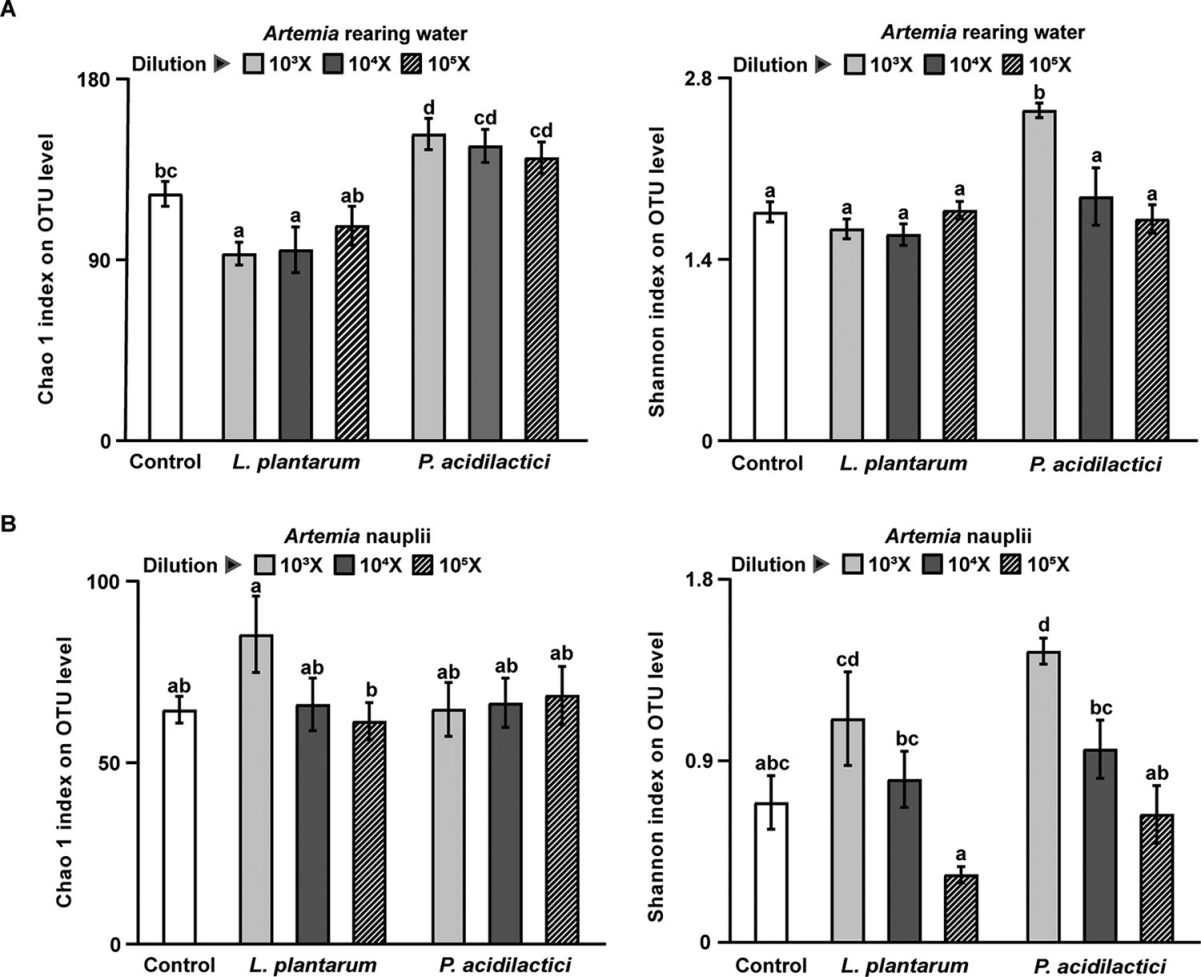
Differences of the Chao 1 and the Shannon indexes from α-diversity analyses on genus abundance of *Artemia* rearing water (A) and Artemia nauplii (B). Statistical significance was evaluated by one-way ANOVA, followed by *post hoc* analysis with Duncan multiple-comparison test. The letters a to d indicate statistically significant differences (*P* < 0.05). Error bars represent the SEM calculated from at least six biological replicates.

Analyses of the changes in microbial community abundance were used to explore the effect of *L. plantarum* and *P. acidilactici* on *Artemia* after hatching. To analyze the microbial community diversity within the samples, α diversity was used to reflect the diversity of microbial communities. The Chao1 and the Shannon indexes from α-diversity analyses were calculated and averaged by experimental groups (Fig. 4). In *Artemia* rearing water, the microbial richness of *L. plantarum* (diluted 10^3^- and 10^4^-fold) treatments significantly decreased (*P* < 0.05) and that of *P. acidilactici* (diluted 10^3^-fold) significantly increased the microbial richness and diversity (*P* < 0.05). Moreover, there was no significant difference in microbial diversity by *L. plantarum* treatments. In Artemia nauplii, the variation trends of microbial diversity were generally similar between the two LAB-treated groups, and the microbial richness of LAB treatments showed no significant difference compared to the control group (*P* > 0.05). Additionally, to exclude the influence of pH values on the microbial community in *Artemia*, the pH values for each treatment were tested in Artemia nauplii during hatching (Table 2). The results showed that there was no significant difference in pH values between the control group and the LAB-treated groups (*P* > 0.05).

**TABLE 2:** The changes of pH values in the hatching of *Artemia* with or without LAB treatments

Group	pH^*a*^
Control	7.24 ± 0.04
*L. plantarum* at dilution of:	
10^3^	7.32 ± 0.07
10^4^	7.30 ± 0.00
10^5^	7.31 ± 0.01
*P. acidilactici* at dilution of:	
10^4^	7.16 ± 0.06
10^5^	7.36 ± 0.02
10^3^	7.30 ± 0.04

a The results are expressed as the mean ± standard error from at three biological replicates.

**FIG 5:**
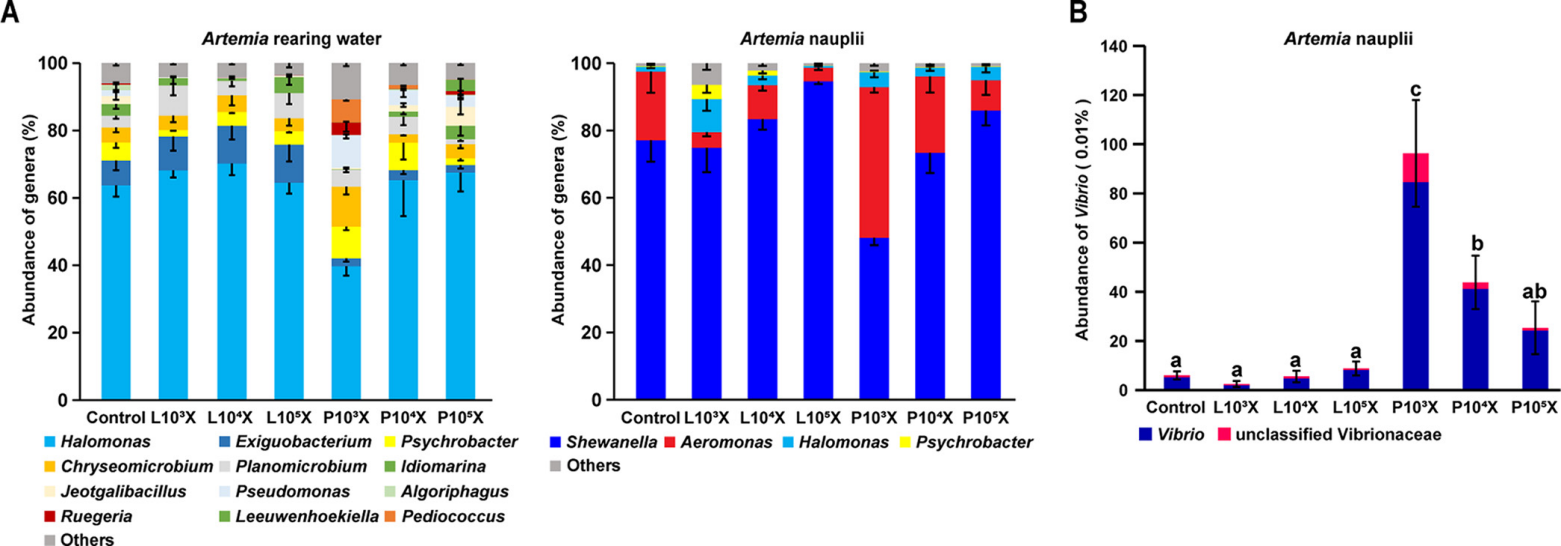
(A) Impacts of the fermentation broths of *L. plantarum* and *P. acidilactici* on abundance of genera in *Artemia* rearing water and Artemia nauplii. Genera with an average of less than 3% detected are not shown. (B) Impacts of the fermentation broths of two LAB isolates on abundance of genus *Vibrio* in Artemia nauplii. Statistical significance was evaluated by one-way ANOVA, followed by *post hoc* analysis with Duncan multiple-comparison test. The letters a to c indicate statistically significant differences (*P* < 0.05). In panels A and B, the fermentation broth of each LAB isolate was diluted 10³-fold (high dosage), 10^4^-fold (intermediate dosage), and 10^5^-fold (low dosage). Error bars represent SEM calculated from at least six biological replicates.

To visualize the variation in abundance of different samples at genus levels, the stack distributions of genera with more than 3% abundance were obtained to visually view the relative abundance and proportions of the genus level (Fig. 5A). For the taxonomy stack distributions at the genus level, the bacterial taxonomic compositions of control, *L. plantarum* (diluted 10³-, 10^4^-, and 10^5^-fold) and *P. acidilactici* (diluted 10³-, 10^4^-, and 10^5^-fold) treatments in *Artemia* rearing water showed a relatively high average abundance of *Halomonas* (63.7% ± 3.36%, 68.14% ± 2.09%, 70.18% ± 3.42%, 64.5% ± 3.21%, 39.73% ± 2.76%, 65.21% ± 10.65%, and 67.51% ± 5.62%, respectively), followed by *Exiguobacterium* (7.37% ± 2.87%, 10.11% ± 4.29%, 11.23% ± 4.08%, 11.29% ± 4.98%, 2.34% ± 1.01%, 3.03% ± 1.16%, and 2.28% ± 1.09%, respectively) and *Psychrobacter* (5.37% ± 2.09%, 1.89% ± 0.24%, 4.08% ± 0.36%, 4.03% ± 0.39%, 9.42% ± 1.07%, 8.17% ± 5%, and 1.93 ± 0.63%, respectively) [see Table S1 at http://www.download-client(bio-marine-scau.com)]. Furthermore, the bacterial taxonomic compositions of control, *L. plantarum* (diluted 10³-, 10^4^-, and 10^5^-fold) and *P. acidilactici* (diluted 10³-, 10^4^-, and 10^5^-fold) treatment in Artemia nauplii showed the average abundance of *Shewanella* (77.07% ± 6.32%, 74.85% ± 7.23%, 83.39% ± 3.12%, 94.63% ± 0.81%, 48.14% ± 2.23%, 73.41% ± 6.01%, and 85.94% ± 4.44%, respectively), followed by *Aeromonas* (20.43% ± 6.29%, 4.66% ± 1.24%, 10.08% ± 1.63%, 4.02% ± 0.65%, 44.79% ± 1.65%, 22.72% ± 4.82%, and 9.04 ± 4.42%, respectively) and *Psychrobacter* (0.19% ± 0.12%, 4.29% ± 2.4%, 1.46% ± 0.89%, 0.09% ± 0.02%, 0.21% ± 0.13%, 0.1% ± 0.06%, and 0.12% ± 0.07%, respectively) [see Table S2 at http://www.bio-marine-scau.com/download/Supplementary%20material_spectrum.zip)]. In addition, *P. acidilactici* (diluted 10³- and 10^4^-fold) significantly enhanced the abundance of *Vibrio* species (from 0.06% to 0.96% and 0.44%, respectively) with a positive dosage-dependent manner in Artemia nauplii (*P* < 0.05). However, there was no significant difference in the abundance of *Vibrio* species by *L. plantarum* treatments (Fig. 5B).

**FIG 6:**
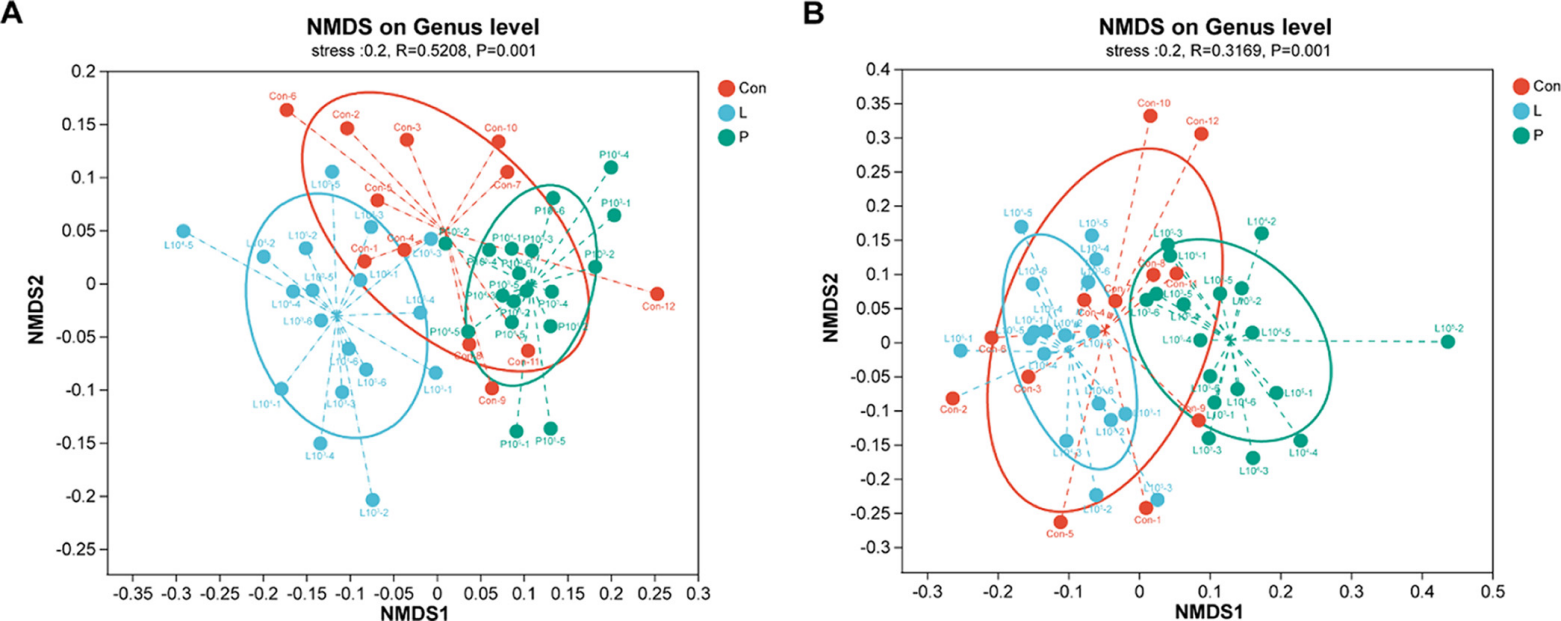
The bacterial microbiota of *Artemia* rearing water (A) and Artemia nauplii (B) among groups were compared using NMDS based on an abundance similarity matrix (unweighted UniFrac distances). The distance between points indicates the similarity of microbiota composition. *R* values in ANOSIM were used to detect overlap in the community (*R* > 0.75, well separated; 0.50 < *R* ≤ 0.75, separated but overlapping; 0.25 < *R* ≤ 0.50, separated but strongly overlapping; *R* ≤ 0.25, barely separated) (92).

Next, to compare the diversity between different ecosystems, nonmetric multidimensional scaling (NMDS) was carried out to analyze the bacterial microbiota in *Artemia* rearing water (Fig. 6A) and Artemia nauplii (Fig. 6B) among groups based on an abundance similarity matrix (unweighted UniFrac distances). Furthermore, a pairwise analysis of similarity (ANOSIM) was used to detect the significant differences in the community of different groups. In the bacterial community of *Artemia* rearing water, the groups of different treatments were well separated between the *L. plantarum* group and *P. acidilactici* group (ANOSIM; *R* = 0.7071; *P* = 0.001) and separated but overlapping between the control group and *L. plantarum* group (ANOSIM; *R* = 0.3307; *P* = 0.001) and between the control group and *P. acidilactici* group (ANOSIM; *R* = 0.4238; *P* = 0.001). In the bacterial community of Artemia nauplii, the groups were barely separated between the control group and *L. plantarum* group (ANOSIM; *R* = 0.136; *P* = 0.021) and separated but overlapping between the *L. plantarum* group and *P. acidilactici* group (ANOSIM; *R* = 4747; *P* = 0.001) and between the control group and *P. acidilactici* group (ANOSIM; *R* = 0.2772; *P* = 0.001).

### Metabolomics of LAB isolates.

**FIG 7:**
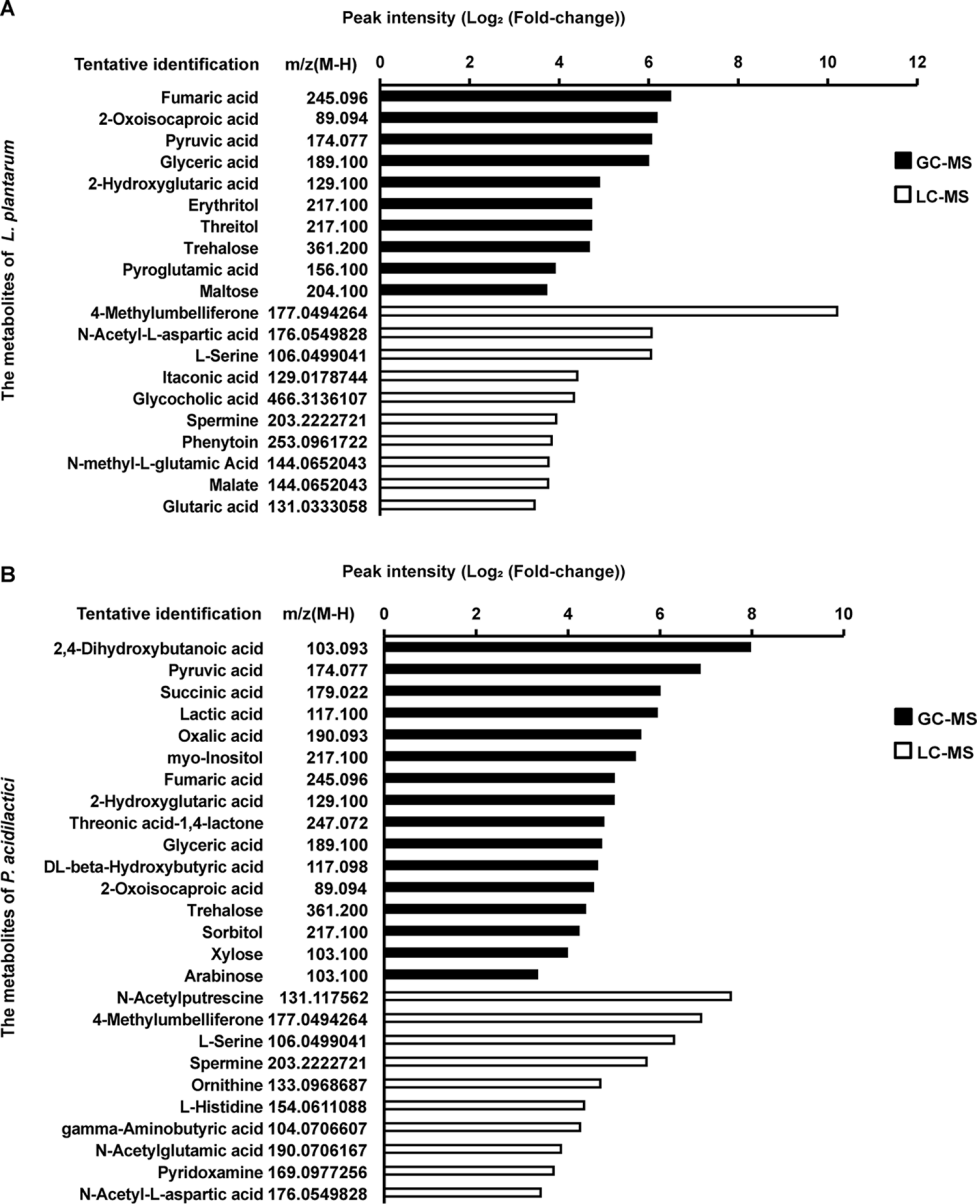
The 20 and 27 most significant abundant metabolites selected from the cell-free supernatant of *L. plantarum* (A) and *P. acidilactici* (B), respectively. The significance of the abundance of these metabolites was determined by the fold change in peak intensity (fold change of >10). The fold change was calculated by comparing the peak intensity of each metabolite in the cell-free supernatant of two LAB isolates to the mean peak intensity of the corresponding compound in the control (MRS broth cultures without inoculation).

The metabolomic analysis of the fermentation broths of *L. plantarum* and *P. acidilactici* by gas chromatography-mass spectrometry (GC-MS) and liquid chromatography-mass spectrometry (LC-MS) are shown in Fig. 7. The 20 bacterial metabolites were selected based on the fold change of peak intensity (fold change of >10) between *L. plantarum* group and control group, and the top 5 of them were fumaric acid, 2-oxoisocarbonic acid, pyruvic acid, glyceric acid, and 2-hydroxyglutaric acid (Fig. 7A). Similarly, we selected 27 bacterial metabolites based on the fold change of peak intensity (fold change of >10) between the *P. acidilactici* group and control group, and the top 5 of them were 2,4-dihydroxybutanoic acid, pyruvic acid, succinic acid, lactic acid, and oxalic acid (Fig. 7B).

### Impacts of pyruvic acid on the *Vibrio* and *Aeromonas* species.

**FIG 8:**
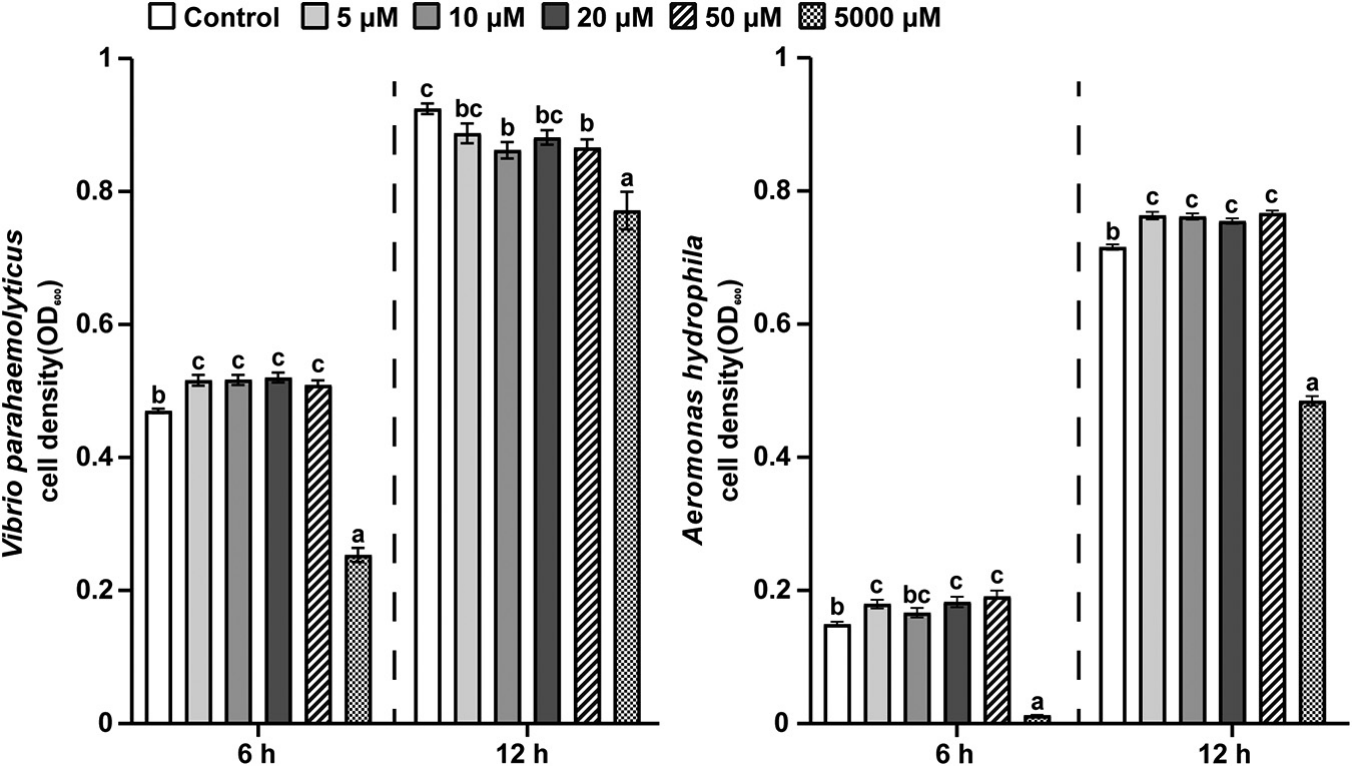
Impacts of the pyruvic acid on the proliferation of the V. parahaemolyticus and A. hydrophila at 6 and 12 h, respectively. The doses of pyruvic acid were 5, 10, 20, 50, and 5,000 μM. Statistical significance was evaluated by one-way ANOVA, followed by *post hoc* analysis with Duncan multiple-comparison test. The letters a to c indicate statistically significant differences (*P* < 0.05). Each point in time was analyzed independently (indicated by dashed lines). Error bars represent the SEM calculated from at three biological replicates.

Based on the LC-MS and GC-MS analyses of the metabolites of the two LAB isolates, pyruvic acid, which was common to both LAB isolates and had a higher yield, was selected for the *in vitro* test. As shown in Fig. 8, the low-dose (5, 10, 20, and 50 μM) pyruvic acid significantly stimulated the proliferation of the A. hydrophila (*P* < 0.05) at 6 and 12 h. Interestingly, the low-dose (5, 10, 20, and 50 μM) pyruvic acid significantly stimulated the proliferation of the V. parahaemolyticus (*P* < 0.05) at 6 h, while the pyruvic acid at 10 and 50 μM significantly inhibited the proliferation of V. parahaemolyticus at 12 h. Moreover, the levels of proliferation of V. parahaemolyticus and A. hydrophila were significantly inhibited by the high-dose pyruvic acid (5,000 μM) treatment at 6 and 12 h. However, the higher acidification of pyruvic acid solution at 5,000 μM might also have an impact on the proliferation of V. parahaemolyticus and A. hydrophila.

## DISCUSSION

Nutrient utilization is considered to be an important factor for probiotics to colonize the host and exert their beneficial effects, and it is relevant to enzyme species and activity (30). Some studies have reported that amylase from *L. plantarum* plays an important role in the gastrointestinal tract of animals (31, 32). In this study, the LAB isolates were positive for amylase activity. Additionally, *L. plantarum* displayed protease activity, which could improve the digestibility of polypeptides and release bioactive peptides with useful activities (33). Moreover, extracellular protease activity is highly correlated with the concentration of lactic acid produced (34), and lactobacilli show antibacterial activity mainly due to the production of lactic acid (35, 36). Swimming and swarming motilities are the phenomena of flagellum-oriented migration on the surface of the culture media, and these motility activities are related to the pathogenicity of pathogens (37), while the LAB isolates showed no swimming and swarming motilities in this study (Table 1).

The antagonistic activity against pathogens is important for evaluating the efficacy of probiotics (1). In this research, the fermentation broths of *L. plantarum* and *P. acidilactici* showed the antagonism against *Aeromonas* and *Vibrio* species, which were the common bacterial pathogens causing diseases in aquatic animals (38, 39) (Fig. 2). Some studies indicated that LAB could decrease the levels of bacterial pathogens by producing antimicrobial substances and competing for adhesion sites and nutrients (40–42). Moreover, our findings showed that the fermentation broths, cells and cell-free supernatant of the two LAB isolates were suppressive to the proliferation of *Vibrio* species (Fig. 2A). Quorum sensing is correlated with the expression of virulence-related genes in many different pathogens (43). In our study, the quorum sensing disrupting activities of the cell-free supernatants of the two LAB isolates against the type strain *V. campbellii* BB120 were strong (Fig. 2B). Previous research has shown dietary supplementation of *L. plantarum* and *P. acidilactici* increased food intake, promoted bile acid secretion, and improved *n*-3 polyunsaturated fatty acids in the fish liver, achieving the purpose of promoting fish growth and improving muscle texture (44). Moreover, the fermentation broth of *L. plantarum* alleviated the symptoms of muscle spoilage in Litopenaeus vannamei by significantly inhibiting the proliferation of Vibrio harveyi in the feed (45). Therefore, *L. plantarum* and *P. acidilactici* are considered potential probiotics.

*Artemia* is used as an excellent model organism for studying diseases of aquatic animals (46), and it can serve as the “carrier of particles” for probiotics to be transferred to crustaceans, fishes, and even to the human body via the food chain (47). However, the amounts of bacterial pathogens (such as genera *Aeromonas* and *Vibrio*) in the Artemia nauplii exponentially increase during hatching and nutrient enrichment (48). Studies showed the probiotic *L. plantarum* protected *Artemia* from a Vibrio anguillarum challenge by enhancing the immune response of *Artemia* and thus contributed to reducing oxidative damage and increasing the survival rate (49). In addition, the extracellular products of *P. acidilactici* inhibited the growth of Vibrio alginolyticus isolated from *Artemia* culture (50). Therefore, our study investigated the impacts of the two candidate probiotics on the bacterial community of *Artemia* rearing water and Artemia nauplii by using 16S rRNA gene amplicon sequencing (Fig. 5A). The results showed that *Halomonas* was the most abundant genus in *Artemia* rearing water, and high-dosage *P. acidilactici* decreased the abundance of *Halomonas* from 63.7% to 39.73% compared to the control group. Studies have well reported that *Halomonas* possesses probiotic potential, including secreting beneficial substances, converting inorganic nitrogen compounds (e.g., ammonia, nitrite, and nitrate), repressing pathogens, and modulating the microbial community (51–53), and the research on its pathogenicity is still scarce at present (54). The abundance of *Psychrobacter* in the control group was 5.37% and was reduced to 1.89%, 4.08%, and 4.03%, respectively, in groups treated with *L. plantarum* at the higher, intermediate, and lower dosages. In contrast, the higher and intermediate dosages of *P. acidilactici* increased the abundances of *Psychrobacter* from 5.37% (control group) to 9.42% and 8.17%, respectively. Hisar et al. first reported that pathogenicity of *Psychrobacter* to rainbow trout (55), but others reported that *Psychrobacter* is a potential probiotic (56). Most Pseudomonas species are common pathogens in animals, plants, and even human beings (57–60). In our study, the abundance of Pseudomonas was decreased from 1.77% (control group) to 0.1%, 0.26%, and 0.11%, respectively, by *L. plantarum* treatments at the higher, intermediate, and lower dosages and was increased to 9.64%, 4.41%, and 3.35%, respectively, by *P. acidilactici* treatments at the higher, intermediate, and lower dosages. Moreover, the abundance of *Exiguobacterium* was increased from 7.37% (control group) to 10.11%, 11.23%, and 11.29%, respectively, by *L. plantarum* treatments at the higher, intermediate, and lower dosages and reduced to 2.34%, 3.03%, and 2.28%, respectively, by *P. acidilactici* treatments at the higher, intermediate, and lower dosages. Several researchers reported *Exiguobacterium* was commonly used for enzyme production, bioremediation, and degradation of toxic substances released into the environment (61–63). Thus, the above results indicated that *L. plantarum* could improve the microecological environment of *Artemia* culture, which was conducive to the colonization of beneficial bacteria in Artemia nauplii and enhanced host defense against pathogens. However, the addition of *P. acidilactici* seemed to have the opposite effect.

Administration of probiotics to *Artemia* cultures is an effective protocol for eliminating pathogens in Artemia nauplii (49, 64). However, a few studies have demonstrated that probiotics are not universally excellent and can even provoke the virulence of pathogens (14, 26). In this study, *Shewanella* was the most abundant genus in Artemia nauplii, and the abundance of *Shewanella* was decreased from 77.07% (control group) to 48.14% by *P. acidilactici* treatment (higher dosage) and enhanced to 94.63 by *L. plantarum* treatment (lower dosage). In addition, the proportion of Shewanella bicestrii in the genus *Shewanella* was about 98% [see Fig. S1A at http://www.download-client(bio-marine-scau.com)], and the current research on S. bicestrii is rarely reported (65, 66). The higher and intermediate dosages of *L. plantarum* increased the abundance of *Psychrobacter* from 0.19% (control group) to 4.29% and 1.46%, respectively, which was the exact opposite of the abundance changes of *Psychrobacter* in *Artemia* rearing water. Therefore, *L. plantarum* might enrich the *Psychrobacter* abundance of *Artemia* rearing water into Artemia nauplii. Sun et al. demonstrated that the potential for *Psychrobacter* as a novel candidate probiotic to improve feed utilization, digestive enzymes, and innate immunity in the grouper Epinephelus coioides (67). Most *Aeromonas* species are conditional bacterial pathogens, the causative agent of fish disease when the aquatic environment has deteriorated (68). A. hydrophila is ubiquitous in freshwater and a classic zoonotic pathogen that can cause gastroenteritis, septicemia, and necrotizing fasciitis (69–71). In our study, a large number of *Aeromonas* species were enriched in Artemia nauplii during hatching, and the proportion of A. hydrophila was about 50% [see Fig. S1B at http://www.download-client(bio-marine-scau.com)]. Interestingly, *L. plantarum* at the higher, intermediate, and lower dosages reduced the abundance of *Aeromonas* from 20.43% (control group) to 4.66%, 10.08%, and 4.02%, respectively, while the higher and lower dosages of *P. acidilactici* increased and decreased the abundance of *Aeromonas*, respectively. Vibriosis is the most prevalent among different bacterial diseases, and *Vibrio* species, representing the most frequent human and aquatic pathogens causing toxigenic cholera and other infections, include V. vulnificus, V. parahaemolyticus, and V. cholerae, etc. (72, 73). Here, *P. acidilactici* significantly increased the abundance of *Vibrio* spp. in Artemia nauplii (Fig. 5B). Thus, *P. acidilactici* should be used with caution. *L. plantarum* prevented colonization of bacterial pathogens in Artemia nauplii; therefore, it might improve the survival rate of *Artemia* and further enhance the commercial value of *Artemia*.

To understand the aforementioned phenomena, the significantly abundant metabolites in the extracellular cell-free supernatant of *L. plantarum* and *P. acidilactici* were, respectively, identified by LC-MS and GC-MS (Fig. 6). Concerning the metabolites of *L. plantarum*, the maltose, itaconic acid, glutaric acid, and malate might be detrimental to *Vibrio* species via direct inhibition or inhibiting quorum sensing of the pathogen (74–79). Furthermore, itaconic acid could inhibit quorum sensing of A. hydrophila and maltose promotes crucian carp survival during a challenge with Aeromonas sobria (76). Therefore, the numerous molecules in the extracellular cell-free supernatant of *L. plantarum* might be inhibitory to *Vibrio* and *Aeromonas* species.

Concerning the metabolites of *P. acidilactici*, lactic acid had a significant antagonistic effect on the *Vibrio* species (80). Arabinose could serve as a signal to induce pellicle production by Vibrio fischeri (81), but others reported that arabinose could inhibit the survival of V. cholerae in aquatic environments (75). Moreover, our results showed that the pyruvic acid was selectively antagonistic to V. parahaemolyticus. Therefore, these metabolites might be attributed to the antagonistic activity of *P. acidilactici* against *Vibrio in vitro* and the synergistic effects *in vivo*. Furthermore, lactic acid had a significant effect on controlling the proliferation of *Aeromonas* species (82, 83). A. hydrophila was equipped with metabolic pathways for utilizing *myo*-inositol, which was essential for the virulence of A. hydrophila (84). Similarly, our data showed that pyruvic acid was conducive to the proliferation of A. hydrophila. These results suggested that different dosages of *P. acidilactici* increased or decreased the abundance of *Aeromonas* in Artemia nauplii, but this hypothesis still needs further verification.

Probiotics have received extensive attention as the mean for pathogen control in aquaculture due to their substantial resources and ability to aid in production of antibacterial substances. However, probiotics might not stably exert a beneficial effect on the host when their prophylactic efficacies are prone to environmental interaction. Therefore, to achieve improvement in seafood safety and human well-being by the beneficial effects of probiotics, careful attention to and in-depth research on the probiotic-pathogen interaction is necessary.

Here, two candidate probiotics, *L. plantarum* and *P. acidilactici*, antagonistic to *Vibrio* and *Aeromonas* species *in vitro*, were tested on *Artemia* under *in vivo* conditions. The findings demonstrated the selective antagonism of the candidate probiotics against the genera *Vibrio* and *Aeromonas* in the bacterial community of Artemia nauplii and further revealed the correlation between the selective effects of the candidate probiotics and their metabolites. These results will provide a basis and reference for the rational use of probiotics and for reducing the inappropriate use of probiotics in aquaculture.

## MATERIALS AND METHODS

### Preparation of Luria-Bertani (LB) broth/agar.

LB0, LB5, LB10, or LB35 broth/agar, consisted of 0.0%, 0.5%, 1%, or 3.5% NaCl, respectively, 1% tryptone (Oxoid, Ltd., Hampshire, United Kingdom), and 0.5% yeast extract (Oxoid, Ltd.), without/with agar, and the above media used in this study were prepared in ultrapure water and autoclaved at 121°C for 15 min before use.

### Isolation and identification of LAB.

All LAB were isolated in crustaceans from the South China Sea. They were purified by streak plating on MRS agar (Guangdong Huankai Microbial Sci. & Tech. Co., Ltd., Guangzhou, Guangdong, China) and incubated at 28°C from 24 to 48 h until single colonies were visible. Each purified isolate was grown in LB0 broth from 24 to 48 h and preserved in 20 to 40% glycerol (Sigma-Aldrich, Co., MO, USA) at −80°C.

After preservation in 20 to 40% glycerol at −80°C, isolates from MRS agar were capable of reviving in LB0 broth. Bacterial DNA was extracted and the 16S rRNA gene was amplified by PCR using 16S primers (5′ forward [F], AGAGTTTGATCCTGGCTCAG; reverse [R], ACGGGCGGTGTGTACA-3′). The PCR amplification system and PCR amplification procedure are shown in Table S3 and Table S4, respectively, at http://www.bio-marine-scau.com/download/Supplementary%20material_spectrum.zip. The PCR product was analyzed by 1% agarose gel electrophoresis at 170 V for 15 min. The product was observed under a gel imaging system using the DL5000 DNA marker as a reference, and LAB strains with well-defined bands, good brightness, and product size of about 1,500 bp were sent to BGI (Shenzhen, Guangdong, China) for 16S rRNA gene sequencing. Fragments were subjected to blastn (85) comparison on the National Centre for Biotechnology Information (NCBI) database website (www.ncbi.nlm.nih.gov/). The identification standard was the critical value of ≥99% identity. To analyze the taxonomy of LAB, the reference sequences of 217 LAB type strains with good sequence quality downloaded from the National Centre for Biotechnology Information (NCBI database website http://www.ncbi.nlm.nih.gov/). A phylogenetic tree was created in MEGA7 based on the 16S rRNA gene sequences (1,200 bp) of two LAB isolates and 217 LAB-type strains and analyzed by ClustalW (86). The evolution distance was Kimura two-parameter mode (87). The nonuniformity of the evolution rate among sites was determined by discrete gamma distribution, the gamma parameter was 0.21, and 2,000 bootstrap replicates were utilized (88).

### Production of LAB fermentation broths.

The fermentation broths of LAB isolates were made by Jiaxing Huida Biotechnology Co., Ltd., using internal fermentation technology. Briefly, the LAB isolates were anaerobically cultured in the MRS broth (Jiaxing Huida Biotechnology Co., Ltd., Zhengjiang, China) for 12 h at a temperature of 35°C, and the number of viable cells in the fermentation broths of *L. plantarum* and *P. acidilactici* were about 2.1 × 10^8^ and 1.05 × 10^9^ CFU mL^−1^, respectively.

### Motility and enzymatic characterization of LAB isolates.

The LAB isolates were inoculated in MRS broth and incubated at 200 rpm at 28°C for 12 to 24 h, the cells of each culture were washed once by centrifugation at 5,000 × *g* for 5 min at room temperature to remove the supernatant, and the cell pellets were resuspended in ultrapure water. After determination of the cell density of each cell suspension with a spectrophotometer (BioTek Synergy HTX multimode reader; BioTek Instruments, Inc., Winooski, VT, USA) measuring the optical density at 600 nm (OD_600_), each cell suspension was adjusted to a cell density of 10^9^ cells mL^−1^.

The motility and enzymatic assays were set up according to the protocols described by Gan and colleagues (26). The swimming and swarming media were prepared in MRS broth supplemented with 0.3% and 0.6% agar, respectively. The amylase indicator medium consisted of 1% wheat powder and LB0 agar. The lipase indicator medium consisted of MRS agar supplemented with 0.6% lecithin from soybean. The protease indicator medium consisted of 1.5% Difco skim milk powder (BD, Franklin Lakes, NJ, USA) and 1.5% agar. The indicator medium of feed degradation consisted of 1% crushed and sieved (through a sieve with a pore size of 425 μm) shrimp feed (type no. 0; Guangdong Yuehai Feed Group, Guangdong, China) supplemented with 1.5% agar. The indicator media for shrimp shell degradation and shrimp meat degradation consisted of 1% shrimp shell powder and 1% shrimp meat powder supplemented with 1.5% agar, respectively. The following method was used to prepare the shrimp shell powder and shrimp meat powder. The shell and muscle of Litopenaeus vannamei were dried in an oven at 105°C for 6 h, and then each material was crushed and passed through a sieve with a pore diameter of 425 μm. The indicator medium for shrimp manure degradation consisted of 1% shrimp manure powder and 1.5% agar. The following method was used to prepare the shrimp manure powder. The shrimp manure powder was first sun-dried and then dried in an oven at 60°C for 24 h, and the matter was crushed and passed through a sieve with a diameter of 425 μm. Then, all culture media were autoclaved at 121°C for 15 min before use. Finally, 2 μL of each cell suspension was spot inoculated in triplicate on MRS plates containing 0.3% agar or 0.6% agar to investigate the swimming and swarming motilities separately, and 3 μL of each cell suspension was spot inoculated in triplicate on other indicator media. The swimming and swarming plates for LAB were inoculated at 26 ± 2°C for 42 and 18 h, respectively. The lipase, protease, and feed agar plates were incubated at 28°C for 7 days, and the amylase, shrimp shell, shrimp meat, and shrimp manure agar plates were incubated at 28°C for 14 days.

### Antagonism of LAB isolates against the *Vibrio* and *Aeromonas* species.

To collect the cell-free supernatant of LAB, the LAB fermentation broth was centrifuged at 5,000 × *g* for 5 min to separate the supernatant from the cell pellets, and then the supernatant was filter sterilized through a 0.22-μm-pore filter (Merck Millipore, Ltd., Co. Cork, Ireland). Moreover, the sterile ultrapure water was added to the remaining cell pellets and mixed well to obtain the cell suspension.

The 10 *Vibrio* strains [see Table S5 at http://www.bio-marine-scau.com/download/Supplementary%20material_spectrum.zip)] were inoculated into LB0 broth at 200 rpm at 28°C for 12 to 24 h. The cells of each culture were washed once by centrifugation at 5,000 × *g* for 5 min at room temperature to remove the supernatant, and the cell pellets were resuspended in 1% saline solution, and then the cell density of each suspension was determined with a spectrophotometer at 600 nm, and each suspension was adjusted to a cell density of 10^9^ cells mL^−1^.

To imitate the dosage range of the already-applied fermentation broth of LAB under the actual agricultural conditions, the cell-free supernatant, cells, and fermentation broths of LAB isolates and the noninoculated MRS broth (control treatment) were diluted 10^4^-, 10^5^-, and 10^6^-fold with LB0 broth. The cell suspension of each *Vibrio* strain was added to each diluted cell-free supernatant, cells and fermentation broths of LAB isolates, and the noninoculated MRS broth to make a culture mixture with an initial density of 10^5^ cells mL^−1^. One hundred microliters of each culture mixture was added into a well of a Costar 96-well cell culture plate (Corning, Inc., Corning, NY, USA). Each treatment was performed in triplicate, and there was no significant difference in pH value of each treatment. The standing cultures were incubated at 28°C, and the absorbance at a wavelength of 600 nm was measured at 0, 6, 9, 12, 15, and 24 h. The proliferation of *Vibrio* treated with a dilution of the cell-free supernatant, cells, and fermentation broths of LAB isolates was judged as inhibited when the mean OD_600_ value of such culture was at least 0.1 U lower than that of the corresponding control culture during the exponential growth phase within 24 h.

In addition, a similar experimental setup was exploited to test the effect of the cell-free supernatant of LAB isolates on the bioluminescence of the type strain *V*. *campbellii* BB120 (ATCC BAA-1116) and the effect of the fermentation broths of LAB isolates on the proliferation of A. hydrophila. The type strain *V*. *campbellii* BB120 was grown in LB35 broth. The cells of *V. campbellii* BB120 were washed once and resuspended in 35‰ saline to a final density of 10^9^ cells mL^−1^. The noninoculated MRS broth (control treatment) and the cell-free supernatant of LAB isolates were diluted 10-, 10^2^-, and 10^3^-fold with 35‰ saline. The cell suspension of *V. campbellii* BB120 was added to each diluted content to make a culture mixture with an initial density of 10^8^ cells mL^−1^, and then the bioluminescence of each culture was measured within 15 min. The bioluminescence of the control treatment was treated as 100% and the other corresponding treatments were accordingly normalized. A. hydrophila was grown in LB5 broth, and the cells of A. hydrophila were washed once and resuspended in 5‰ saline to a final density of 10^9^ cells mL^−1^. The fermentation broths of LAB isolates were diluted 10^2^-, 10^3^-, 10^4^-, and 10^5^-fold with LB5 broth. The non-LAB treatment was the control, and the cell suspension was added to each diluted content to make a culture mixture with an initial density of 10^6^ cells mL^−1^. The standing cultures were incubated at 28°C and the absorbance at a wavelength of 600 nm was measured at 0, 6, 12, and 24 h.

### Impacts of LAB isolates on TCBS-cultured bacteria in *Artemia*.

A total of 0.5 g of *Artemia* cysts (Huanhai brine shrimp egg factory of Haixing County, Cangzhou, Hebei, China) was hatched in 400 mL sterile artificial seawater (pH 9) containing 3.5% seawater crystal (Yanzhibao, Guangzhou, Guangdong, China) at a temperature of 26 ± 2°C with continuous oxygenation and constant illumination (~2,000 lx), followed by addition of 4, 40, and 400 μL of fermentation broths of LAB isolates when the *Artemia* cysts were hatched at 18 h. The non-LAB treatment was the control, and each treatment was set up in triplicate. After *Artemia* cysts were hatched for 22 h, the *Artemia* rearing water and Artemia nauplii cysts were collected, and the pH values of *Artemia* rearing water were determined. Finally, 20 Artemia nauplii cysts from each replication group were gathered, washed three times with sterile artificial seawater, placed into a sterile 1.5-mL Eppendorf tube containing 80 or 100 μL sterile artificial seawater and two sterile glass beads with a diameter of 3 mm, and then shaken for 3 to 5 min to prepare *Artemia* suspension.

The *Artemia* suspension and *Artemia* rearing water were diluted with sterile artificial seawater in a 10-fold gradient, 5-μL spots from each gradient were placed on the *Vibrio* semiselective medium TCBS (thiosulfate-citrate-bile salts-sucrose) agar (Guangdong Huankai Microbial Sci. & Technology Co., Ltd., Guangzhou, Guangdong, China), and each agar plate was spot inoculated three times. After drying, the agar plates were placed in an incubator at 28°C, and then they were taken out for colony counting after 18 h.

### Impacts of LAB isolates on microbial community abundance in *Artemia*.

A total of 2.5 g of *Artemia* cysts was hatched in 2 L sterile artificial seawater (pH 9) containing 3.5% seawater crystal at a temperature of 26 ± 2°C with continuous oxygenation and constant illumination (~2,000 lx), followed by addition of 0.02-, 0.2-, and 2-mL fermentation broths of LAB isolates when the *Artemia* cysts were hatched at 18 h. The non-LAB treatment was the control, and six biological replicates were set up. After the *Artemia* cysts were hatched at 24 h, the *Artemia* rearing water was collected, filtered through a 0.22-μm-pore filter membrane (Pall Corporation, NY, USA), and stored in a −80°C refrigerator.

The hatching of sterile *Artemia* was carried out according to the protocols described by Defoirdt and colleagues (46). Briefly, 5 g of *Artemia* cysts was hydrated in 400 mL sterile artificial seawater for 1 h. Totals of 14.67 mL of NaOH (32%) and 222.22 mL of NaOCl (50%) were added to the hydrated *Artemia* cyst suspension, and the decapsulation was stopped after 2 min by adding 311.11 mL Na_2_S_2_O_3_ (10g L^−1^). During the reaction, 0.22-μm-pore-filtered aeration was provided. The decapsulated cysts were washed three times with sterile artificial seawater, and the *Artemia* cysts were divided into 12 equal parts and suspended in 500-mL glass bottles containing 400 mL artificial seawater, and then the cysts were hatched at a temperature of 26 ± 2°C with continuous oxygenation and constant illumination (~2,000 lx). After the *Artemia* cysts were hatched for 18 h, the fermentation broths of LAB isolates were added at 4, 40, and 400 μL in the glass bottles described above. The non-LAB treatment was the control, and six biological replicates were set for each treatment. The *Artemia* suspension was poured into a pear-shaped separatory funnel with a capacity of 1,000 mL when *Artemia* cysts were hatched for 24 h, and then the Artemia nauplii cysts were collected at the bottom of the pear-shaped separatory funnel using a 0.40-μm-pore Falcon cell strainer (Corning, Inc., Corning, NY, USA), washed three times with sterile artificial seawater, and stored in a −80°C refrigerator.

DNA extraction, PCR amplification of the 16S rRNA gene, Illumina MiSeq sequencing, and processing of the sequencing data were performed by Majorbio Bio-Pharm Technology Co. Ltd. (Shanghai, China) according to the protocols described by Wang and colleagues (89). The sequencing raw reads were filtered to obtain high-quality clean reads, which were merged as raw tags using FLASH (v.1.2.11), with a minimum overlap of 10 bp and mismatch error rates of 2%. Then, raw tags were filtered by the QIIME (v.1.9.1) pipeline under specific filtering conditions to obtain high-quality clean tags. The effective tags were merged and clustered into operational taxonomic units (OTUs) with 97% sequence similarity using the UPARSE pipeline (90). The tag sequence with the highest abundance was selected as the representative sequence within each cluster, and a Venn analysis was performed in R to identify unique and common OTUs. The Chao1 and the Shannon indexes from α-diversity analysis were calculated in QIIME (v.1.9.1). Nonmetric multidimensional scaling (NMDS) ordination based on the unweighted UniFrac metric was performed, and analysis of similarities (ANOSIM) using the unweighted-unifrac metric distance was carried out.

### Metabolomics of LAB isolates.

The fermentation broths of LAB isolates were supplied by Jiaxing Huida Biotechnology Co., Ltd., and the cell-free supernatant was prepared according to the aforementioned method and stored at −80°C before further processing. The MRS medium was included as a control for determination of the background. Metabolite extraction, liquid chromatography-mass spectrometry (LC-MS) analyses, and gas chromatography-mass spectrometry (GC-MS) analyses were conducted by Bionovogene (Suzhou, Jiangsu, China) according to the protocols described by Gan and colleagues (26).

### The impacts of pyruvic acid on the *Vibrio* and *Aeromonas* species.

The method for culturing V. parahaemolyticus and A. hydrophila was performed according to the experimental protocol described above. The pyruvic acid (Sigma-Aldrich, Co., MO, USA) was adjusted to 5, 10, 20, 50, and 5,000 μM with LB10. The cell suspension of V. parahaemolyticus was added to each concentration of pyruvic acid and LB10 (control treatment) to make a culture mixture with an initial density of 10^5^ cells mL^−1^. One hundred microliters of each culture mixture was added to a well of a Costar 96-well cell culture plate. The standing cultures were incubated at 28°C, and the absorbance at a wavelength of 600 nm was measured at 0, 6, and 12 h. A similar experimental protocol was exploited to test the impacts of the pyruvic acid on the proliferation of A. hydrophila: each concentration of pyruvic acid was adjusted with LB5 broth accordingly, and the LB5 treatment was the control.

### Statistical analysis.

Each treatment was carried out with at least three valid replicates, and results were expressed as mean values indicating standard error. Statistical comparisons were made by one-way analysis of variance (ANOVA) with *post hoc* Duncan analysis (SPSS Statistic 26.0 software; IBM, USA). Statistically significant differences were considered at the 0.05 level.

### Data availability.

Raw read data have been submitted to the NCBI Sequence Read Archive (SRA) under accession no. SRR23379335 to SRR23379430 (BioProject no. PRJNA932970). Good-quality sequences of the isolates in this article have been deposited in GenBank. The accession no. for the 16S rRNA gene sequences of the *L. plantarum* and *P. acidilactici* are OQ410491 and OQ410492.
